# Uterine Fibroid-Associated Massive Pulmonary Embolism: A Report of Two Cases

**DOI:** 10.7759/cureus.62436

**Published:** 2024-06-15

**Authors:** Donclair Brown, Natasha Ghalib, Jovanna Wu, Ambreen Shahzadi, Tamara Simpson, Aayushi Sharma, Vishal Reddy Bejugam, Kristin Soodhan, Rosy Thachil

**Affiliations:** 1 Internal Medicine, North Central Bronx, Jacobi Medical Center, New York, USA; 2 Internal Medicine, Montefiore Medical Center, New York, USA; 3 Biological Sciences, Stuyvesant High School, New York, USA; 4 Internal Medicine, Icahn School of Medicine at Mount Sinai, New York, USA; 5 Internal Medicine, University Hospital of the West Indies, Kingston, JAM; 6 Cardiology, Elmhurst Hospital, Icahn School of Medicine at Mount Sinai, New York, USA

**Keywords:** thrombophilia, women's health, leiomyoma, uterine fibroid, pulmonary embolism

## Abstract

This report details cases of uterine fibroid-associated deep vein thrombosis leading to massive pulmonary embolism, as well as the likely associated physiology. Two women, aged 33 and 37, presented with fibroid-associated pulmonary embolism. They both had large uterine sizes and no underlying thrombophilia. Case 1 had an uncomplicated course, whereas Case 2 had a course complicated by cardiac arrest and prolonged recovery. The presence of fibroids enhances coagulation and platelet adhesion. Mechanical compression also plays a role in predisposing to thrombosis. There may be a role for preoperative screening, especially in those with an elevated estimated uterine weight.

## Introduction

Uterine fibroids (UF), diagnosed in as many as 70% of Caucasian women and approaching 80% in African American women, are characterized by heavy menstrual bleeding, dysmenorrhea, and less frequent compressive symptoms such as constipation and urinary retention. Recent studies reveal a notable additional concern: a possible connection between uterine fibroids and acute venous thromboembolism (VTE) [1,2]. This is thought to be due to direct vascular compression in addition to an increase in procoagulant factors induced by the presence of uterine fibroids. Heavy menstrual bleeding is also a feature of this population, and this makes the use of anticoagulation difficult. Patient selection will be critical to maximize the benefit and decrease the risks of any interventions needed to decrease this VTE risk. This report details cases of UF-associated deep vein thrombosis leading to massive pulmonary embolism and the likely associated pathophysiology. We also highlight the possible role of preoperative screening in this population.

This article was previously presented as a meeting abstract at the 2024 ACC Annual Scientific Meeting on April 7, 2024.

## Case presentation

Case 1

**Figure 1:**
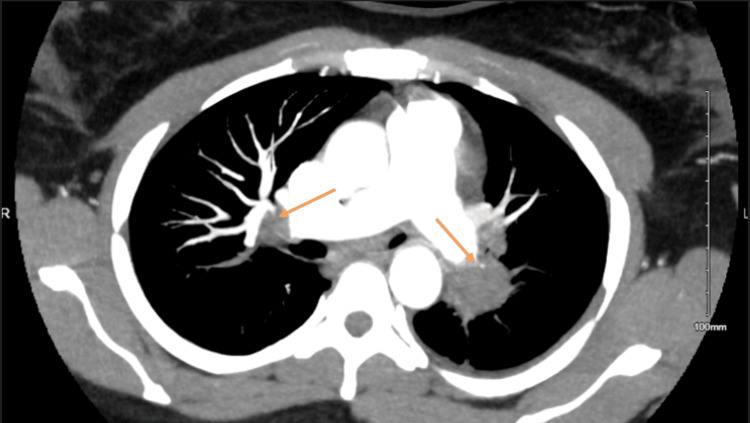
CT-PA demonstrating bilateral PE (orange arrows) CT-PA: computerized tomography of pulmonary arteries; PE: pulmonary embolism

**Figure 2:**
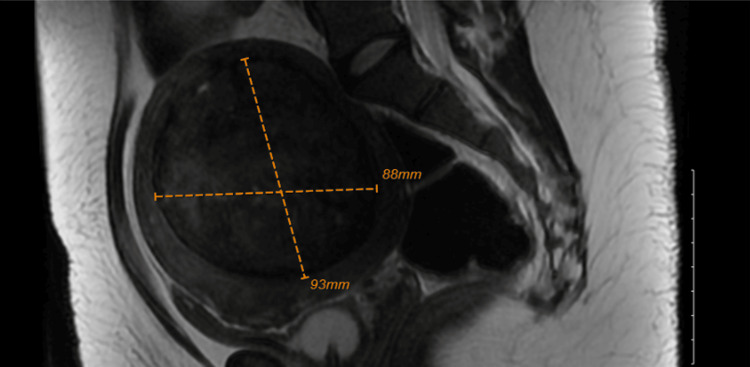
MRI pelvis demonstrating largest intramural fibroid

A 33-year-old woman with known large uterine fibroids presented to the emergency department for recurrent urinary retention. She had no personal or family history of thrombophilia and used no oral contraceptives. In the emergency room (ED), she was tachycardic to 120 per minute; further lab evaluation demonstrated an elevated D-dimer (2,397 ng/mL, normal<=230 ng/mL) and pro b-type natriuretic peptide (pro-BNP) (1,362 pg/mL, normal<=125 pg/mL). Table 1 displays the other lab findings. Computerized tomography of the pulmonary arteries (CT-PA) was done and demonstrated bilateral main pulmonary artery emboli (Figure 1) with evidence of right heart strain and a lingular infarct. There was also an incidental 4.3 cm anterior mediastinal mass, with an associated mass effect on the trachea. Enoxaparin was started, and she was admitted to the cardiac intensive care unit (CICU). Hemodynamics remained stable during her stay in the CICU, and a transthoracic echocardiogram (TTE) confirmed a right heart strain with a positive McConnell's sign. Catheter-directed thrombolysis was performed, with a resulting improvement in right ventricular function. A pelvic MRI was done before discharge and demonstrated two posterior intramural fibroids (9.3 x 8.3 x 7.7 cm and 4.2 x 4 x 3 cm) and one anterior intramural fibroid (1.2 x 0.8 x 1 cm) (Figure 2) with compression of bilateral external iliac veins in addition to right femoral and external iliac deep venous thrombosis (DVT). She was further transitioned to a maintenance dose of apixaban before discharge. The mediastinal mass was later found to be a goiter with retrosternal extension, and her thrombophilia workup was negative (Table 2).

**Table 1:** Selected laboratory values during hospital stay of Case 1 WBC: white blood cell; Na+: sodium; K+: potassium; CI: chloride; CO2: carbon dioxide; BUN: blood urea nitrogen; PT: prothrombin time; INR: international normalized ratio; aPTT: activated partial thromboplastin time; proBNP: pro b-type natriuretic peptide

	Day 0	Day 5	Day 10	Pre-discharge
Hemoglobin (g/dL) (13.5-17.5)	11.9	10.8	11.0	11.3
WBC (/nL)(3.9-10.6)	13.94	10.79	10.54	8.51
Platelets (/nL)(150-440)	428	383	653	522
Na+ (mEq/L) (135-145)	130	137	135	140
K+ (mEq/L) (3.5-5.0)	4.2	4.6	5.1	4.5
Cl- (mEq/L)(98-108)	94	96	97	103
CO2 (mEq/L)(24-30)	24.4	27.4	24.3	22.2
BUN (mg/dL)(5-26)	9	11	12	20
Creatinine (mg/dL)(0.5-0.9)	1.0	0.7	0.8	0.9
PT(s)(9.4-12.5)	16.6		15.8	
INR (0.8-1.1)	1.4		1.4	
aPTT (s)(25.1-36.5)	48.9	69.7	63.5	
D-dimer ng/mL(<=230)	2,397			
Trop T (ug/L)(0.0-0.09)	<0.01	<0.01		
proBNP (pg/mL)(1-125)	1,362	219		

**Table 2:** Thrombophilia workup for Case 1 dRVVT: dilute Russell's viper venom time; APTT: activated partial thromboplastin time

	Findings
Anti-cardiolipin Ab screen	Negative
Silica clotting time	Lupus anticoagulant negative
dRVVT(ratio 0-1.21)	1.28 (LA pos)
APTT 50/50 mix	Non-specific inhibitor
Beta 2 glycoprotein Ab screen	Negative
Factor V assay (%) (60-130)	114 (normal)
Phospholipase A2	Negative
Antithrombin III assay (%)(75-125)	99 (normal)

Case 2

**Figure 3:**
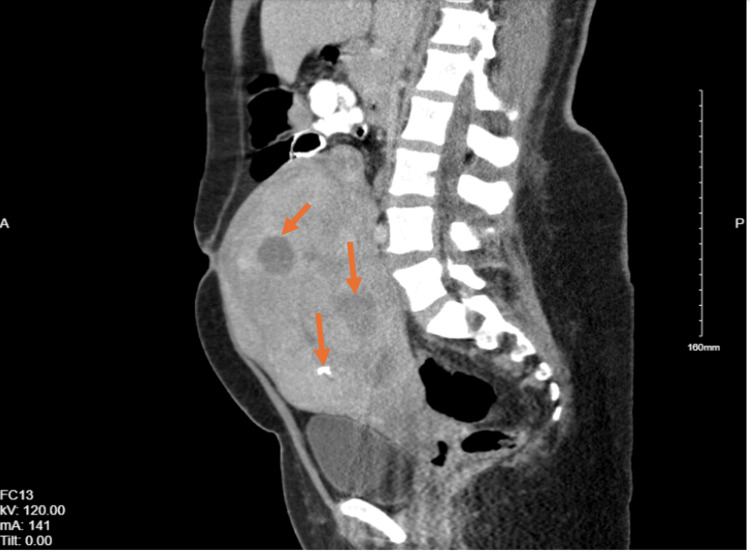
CT-PA demonstrating severely enlarged uterus(10x18x15cm) with multiple fibroids (orange arrows ) CT-PA: computerized tomography of pulmonary arteries

**Figure 4:**
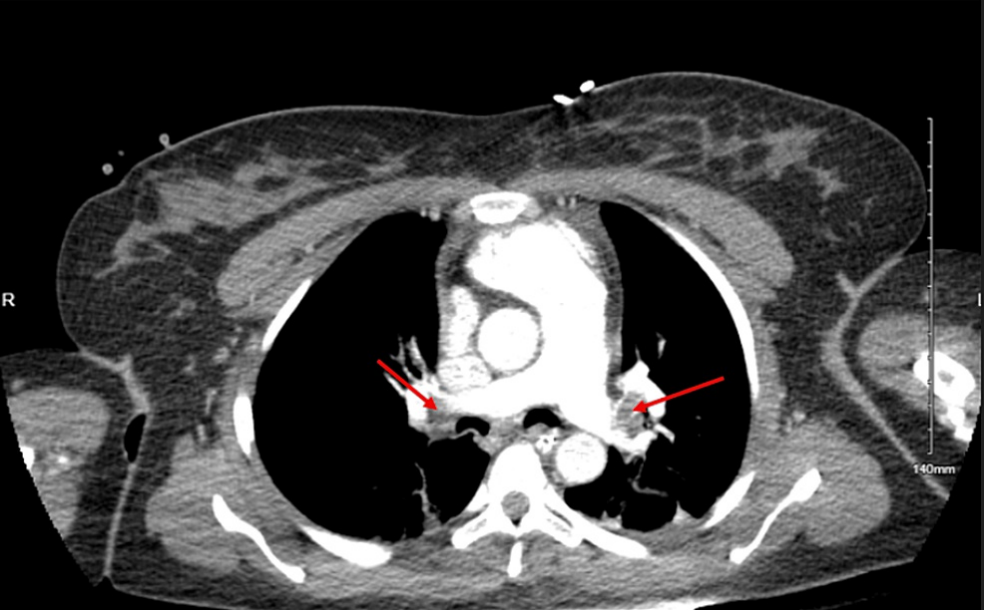
CT chest with contrast demonstrating bilateral embolisms (red arrows) with grossly dilated pulmonary artery

A 37-year-old woman with a known history of uterine fibroids (Figure 3) and iron deficiency anemia was admitted for elective myomectomy. A myomectomy was done successfully with the removal of 17 fibroids. Within the first post-op day, she developed acute severe hypoxia and tachycardia with elevated D-dimer (15,686 ng/mL, normal<=230 ng/mL). Table 3 displays the other lab findings. She was found to have massive bilateral pulmonary embolism (PE), a suspected right atrial clot, and a possible patent foramen ovale on CTPA (Figure 4). Bedside TTE was done with a positive McConnell's sign and was also suggestive of a persistent right atrial clot in transit. Full-dose systemic fibrinolysis was done, and repeat TTE demonstrated persistence of the clot. The decision was then made to start extracorporeal membrane oxygenation (ECMO). During intubation prior to ECMO, she suffered a pulseless electrical activity arrest with a return of spontaneous circulation after 15 minutes. The hospital course was further complicated by severe uterine bleeding necessitating uterine artery embolization. Mechanical thrombectomy was done, and an inferior vena cava (IVC) filter was also placed in light of persistently falling hemoglobin and concern for occult bleeds, which precluded further anticoagulation. The thrombophilia workup (Table 4) was found to be negative. She was eventually discharged with a tracheostomy to a skilled nursing facility. 

**Table 3:** Selected laboratory values during hospital stay of Case 2 WBC: white blood cell; Na+: sodium; K+: potassium; CI: chloride; CO2: carbon dioxide; BUN: blood urea nitrogen; PT: prothrombin time; INR: international normalized ratio; aPTT: activated partial thromboplastin time; proBNP: pro b-type natriuretic peptide

	Day 0	Day 2	Day 10	Pre-discharge
Hemoglobin (g/dL) (12-16)	9.8	7.0	10.8	10.6
WBC (/nL)(3.5-11)	11.17	4.94	14.82	7.00
Platelets (/nL) (150-440)	254	72	102	363
Na+ (mEq/L)(135-145)	146	144	152	142
K+ (mEq/L) (3.5-5.0)	3.6	6.0	3.5	3.6
Cl- (mEq/L)(98-108)	101	114	137	95
CO2 (mEq/L)(22-29)	18	24	21	32
BUN (mg/dL)(6-20)	22	19	36	68
Creatinine (mg/dL)(0.5-1.4)	1.3	1.2	0.8	0.9
INR (0.8-1.1)	1.4	1.1	1.2	-
aPTT(s) (27.8-37.3)	68.8	25.0	28	-
Fibrinogen (mg/dL) (152 - 427)	83.0(L)	374	242	-
D-dimer ng/mL (<=230)	15,686(H)	3,463(H)	39,038(H)	-
Trop T (ug/L)(0.0-0.09)	<0.01	-	-	-
proBNP (pg/mL)(1-125)	99.3	-	-	81.8

**Table 4:** Thrombophilia and HIT workup for Case 2 dRVVT: dilute Russell's viper venom time; APTT: activated partial thromboplastin time; HIT: heparin induced thrombocytopenia

	Findings
Heparin-PF4 Ab	Negative
Serotonin releasing assay	Negative
Anti-cardiolipin Ab screen	Negative
dRVVT (ratio 0-1.21)	0.96 (LA negative)
Silica clotting time	Lupus anticoagulant negative
Beta 2 glycoprotein Ab screen	Negative

## Discussion

Both cases highlight the potential risk of clinically significant VTE in UF patients.

Fibroids increase venous stasis through direct compression. Large fibroids can exert a mass effect on the pelvic venous system and IVC, thus causing stasis in the pelvis and lower extremities. Venous stasis can also be caused by immobility or decreased activity during periods of heavy bleeding or debilitating pain and in the perioperative period for myomectomies and hysterectomies. 

Endothelial injury may occur similarly to that seen in May-Thurner syndrome, where chronic irritation of the endothelium by the overlying compression causes the formation of venous fibrous bands, or 'spurs,' which promote clot formation. However, reported discordance between the location of DVT and the location of the fibroid suggests that mechanical compression is not the only factor at play in predisposing to thrombosis [1-3].

Fibroids also upregulate the expression of type 1 basic fibroblast growth factor receptors and thrombospondin 1, both of which are thought to contribute to enhanced coagulation and platelet adhesion [4-6]. This risk is also likely augmented by the reactive thrombocytosis induced by blood loss and resultant anemia [7].

Age is also a risk factor, as the highest prevalence of uterine fibroids is seen in the 35-44-year-old age group, with a decline in prevalence seen in the post-menopausal age group [8].

In Case 1, we see that our patient had a weakly positive diluted Russell viper venom time (dRVVT) test and a positive mixing study. It’s likely this was a false-positive result due to the initiation of enoxaparin before the tests were conducted. The suspicion of a false-positive result is further heightened as other antiphospholipid markers are negative.

Case 2 presents a scenario in which a patient, who already had uterine fibroids, experienced massive bilateral pulmonary embolism shortly after undergoing myomectomy. Both of these cases lead us to hypothesize that there may be a role for preoperative VTE screening in patients with uterine fibroids. It would not be feasible to screen all patients with uterine fibroids given that close to 70% of reproductive-age women globally will develop fibroids [8]. These investigations would involve additional blood work and the use of imaging modalities such as ultrasound and a possible CT with radiation exposure. 

How do we decide which patients would benefit maximally from screening? Estimated uterine weight may be of use in deciding screening eligibility, as a weight of 1 kg or greater was associated with an over 300% increase in DVT occurrence [9]. 

Estimating uterine weight through non-surgical imaging is challenging but can be approximated using methods such as ultrasound and magnetic resonance imaging. Ultrasound allows for uterine volume calculations based on length, width, and height measurements, with three-dimensional ultrasound potentially providing more accurate assessments. Similarly, MRI offers volumetric assessments and 3D reconstructions for uterine volume calculation. These non-invasive approaches serve as estimations rather than precise measurements of uterine weight, which is conventionally determined through surgical removal and direct weighing [10,11].

VTE surveillance poses its challenges, as discordance may exist between the site of the fibroid and the site of thrombosis.

## Conclusions

The results from observational studies do not definitively establish a cause-and-effect relationship between fibroids and DVT, and we still lack a clear consensus in the medical literature. Therefore, the need for further prospective studies cannot be overstated. Understanding this crucial relationship will enable the medical community to more confidently estimate the thrombotic risk in this population and develop screening strategies and interventions to mitigate this risk. 
